# Exceptionally high-affinity Ras binders that remodel its effector domain

**DOI:** 10.1074/jbc.M117.816348

**Published:** 2017-12-27

**Authors:** John H. McGee, So Youn Shim, Seung-Joo Lee, Paige K. Swanson, Sam Y. Jiang, Michael A. Durney, Gregory L. Verdine

**Affiliations:** From the Departments of ‡ Molecular and Cellular Biology,; § Stem Cell and Regenerative Biology, and; ‖ Chemistry and Chemical Biology, Harvard University and Harvard Medical School, Cambridge, Massachusetts 02138 and; ¶ FOG Pharmaceuticals, Cambridge, Massachusetts 02140

**Keywords:** cancer, directed evolution, peptides, Ras protein, X-ray crystallography

## Abstract

The Ras proteins are aberrantly activated in a wide range of human cancers, often endowing tumors with aggressive properties and resistance to therapy. Decades of effort to develop direct Ras inhibitors for clinical use have thus far failed, largely because of a lack of adequate small-molecule–binding pockets on the Ras surface. Here, we report the discovery of Ras-binding miniproteins from a naïve library and their evolution to afford versions with midpicomolar affinity to Ras. A series of biochemical experiments indicated that these miniproteins bind to the Ras effector domain as dimers, and high-resolution crystal structures revealed that these miniprotein dimers bind Ras in an unprecedented mode in which the Ras effector domain is remodeled to expose an extended pocket that connects two isolated pockets previously found to engage small-molecule ligands. We also report a Ras point mutant that stabilizes the protein in the open conformation trapped by these miniproteins. These findings provide new tools for studying Ras structure and function and present opportunities for the development of both miniprotein and small-molecule inhibitors that directly target the Ras proteins.

## Introduction

The Ras proteins are master regulators of cell proliferation and survival that transduce growth stimuli emanating from cell-surface receptors to cytoplasmic kinases responsible for orchestrating a comprehensive cell division program (1). Under normal physiologic conditions, Ras proteins play a prominent role in a broad range of growth-related processes, whereas variant Ras proteins arising via acquired mutations in the *RAS* genes act as powerful drivers of oncogenesis (2, 3). Activating mutations in *RAS* genes are found in greater than one-third of all neoplasms across a broad range of tumor types and are associated with disease aggressiveness and poor responses to treatment (4, 5). Such mutations are sufficient to initiate tumor growth in mice (6), and acute withdrawal of Ras expression in inducible models leads to apoptosis and regression of established tumors (7).

Ras proteins are cytosolic, membrane-bound GTPases that bind guanine nucleotides and are regulated by their nucleotide state: guanosine triphosphate (GTP)–bound Ras adopts a conformation that binds Ras effector proteins with high affinity (8), leading to their activation and downstream signaling, whereas guanosine diphosphate (GDP)–bound Ras adopts a conformation that cannot bind effectors and thus is inactive (9). The nucleotide state is controlled by a combination of slow, spontaneous GTP hydrolysis with accelerated GTP hydrolysis catalyzed by GTPase-activating proteins and nucleotide exchange stimulated by guanine nucleotide exchange factors. Both spontaneous and GTPase-activating protein–catalyzed GTP hydrolysis can by impaired by point mutations at a number of residues in Ras, which leads to constitutive signaling. The three canonical Ras proteins (KRas, NRas, and HRas) are mutated in a broad range of human cancers with *KRAS*, *NRAS*, and *HRAS* mutations accounting for ∼22, ∼8, and ∼3% of all cancers, respectively (4).

The prevalence of Ras-driven malignancies, taken together with evidence that mutant *RAS* tumors are “addicted” to chronic Ras pathway activation (10, 11), has lent particular urgency to efforts to develop drugs that target Ras proteins directly. Despite intensive efforts spanning more than two decades, no such therapeutic agents have entered human clinical trials, the principal difficulty being that the Ras proteins are intracellular and thus outside the reach of protein-based therapeutics, and they lack an accessible hydrophobic pocket invariably required for targeting by cell-penetrant small molecules. Although Ras proteins do contain a pocket that is capable of binding GTP and GDP, these endogenous cofactors bind Ras with picomolar affinity and are present at millimolar concentrations in the cell, rendering effectively impossible any competition by an exogenously added small molecule. The remainder of the Ras protein surface is relatively flat and hence lacks pockets suitable for high-affinity engagement by small molecules (12).

Recent advances in the understanding of Ras structural biology and the advent of fragment-based drug discovery methodology have led to a resurgence of activity aimed at discovering small-molecule direct-acting Ras antagonists. Structural studies have revealed conformational plasticity of the Ras protein surface, suggesting the possibility that cryptic, targetable pockets might exist. Several recent studies have confirmed the existence of such cryptic pockets and metal-binding sites in various isoforms and mutant states of Ras. Solution nuclear magnetic resonance (NMR) and computational screens have yielded small molecules that engage the outer surface of the Ras switch II region (13–16), and others have exploited covalent adduction to develop antagonists selective for the G12C mutant form of KRas (17, 18), which revealed a cryptic binding pocket behind Ras switch II. Several groups have also developed peptidomimetic Ras inhibitors either based on α-helical segments of known Ras-binding proteins or by screening peptide libraries (19, 20).

These recent non-traditional approaches toward targeting Ras by small molecules have shown some promise, but none have yet demonstrated the degree of affinity and specificity that will ultimately be required for human administration. Here, we report our initial findings on an alternative approach, one that exploits directed evolution to discover miniproteins having extraordinarily high affinity for the most functionally relevant surface of Ras, the effector domain. These tool ligands should prove useful in biochemical studies of Ras, and they reveal new opportunities for both small-molecule and cell-penetrating miniprotein approaches toward direct Ras targeting.

## Results

### Discovery and evolution of Ras-binding miniproteins

**Figure 1.**
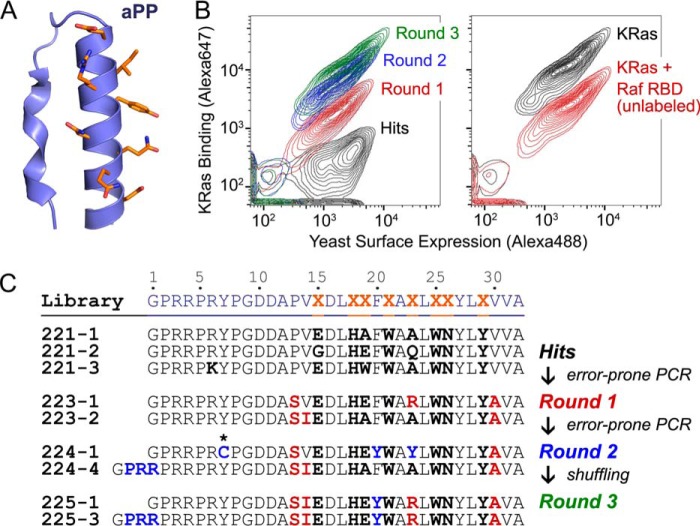
**Discovery of miniproteins that bind the Ras proteins and block the Ras-Raf interaction.** *A*, structure of the aPP scaffold used as the basis of the library with randomized residues shown in *orange* (Protein Data Bank code 1PPT). *B*, *left panel*, FACS plots of representative isolates from each round when incubated with 250 nm KRas(G12V)-GppNHp-Alexa Fluor 647. *Right panel*, FACS of the 225-3 clone in the presence of 250 nm KRas(G12V)-GppNHp-Alexa Fluor 647 with or without 5 μm unlabeled B-Raf RBD. *C*, sequences of library members isolated after each round of enrichment. The *asterisk* (*) indicates Y7C mutation. The initial library screen (to obtain hits) was performed twice and afforded similar hit sequences; the remaining evolution rounds were performed once.

In an effort to develop miniprotein inhibitors of the Ras effector domain, we initiated a screening campaign to identify novel Ras binders, selecting a number of small, conformationally stabilized miniprotein scaffolds that we randomized at a subset of surface-exposed residues. We performed the screens to identify KRas-binding miniproteins using yeast surface display (YSD)^6^ (21) in which miniprotein variants were expressed on the yeast surface and binders to labeled KRas protein were isolated by fluorescence-activated cell sorting (FACS). We obtained three screening hits from a library based on the avian pancreatic polypeptide (aPP), a miniprotein that Schepartz and co-workers (22, 23) have developed as a helical display scaffold for molecular recognition and as a vector for cellular delivery. aPP comprises an N-terminal type II polyproline (PPII) helix joined by a short loop to a C-terminal α-helix, which it stabilizes through hydrophobic interactions (Fig. 1*A*) (24). The particular scaffold that afforded these hits (Fig. 1*B*) was a 32-amino acid aPP mutant containing eight randomized positions on the outward face of the α-helix. The three hits shared a consensus of identical residues at five of these positions (His-18, Trp-21, Trp-25, Asn-26, and Tyr-29) (Fig. 1*C*).

To further improve the KRas binding affinity of the hit miniproteins, we performed three rounds of directed evolution, beginning with random mutagenesis of the hit sequences followed by YSD at a lower concentration of KRas target. Of note, Round 2 afforded improvements in KRas binding due to two unexpected changes in the sequences: a Y7C mutation and a 9-base pair extension encoding a Pro-Arg-Arg (PRR) extension on the N terminus of the miniprotein, the latter caused most likely by primer slipping during PCR amplification (Fig. 1, *B* and *C*). The Y7C mutation was surprising because the consensus residues from the original hits and the mutations from Round 1 suggested that the miniprotein-Ras interaction occurred on the opposite side of the miniprotein from position 7. Wary of a binding mechanism that involved disulfide formation to an exposed KRas cysteine such as Cys-118, which would not be favored under the reducing conditions of mammalian cells, we chose to remove this mutation from our subsequent libraries. For Round 3 of directed evolution, we shuffled the residues from Rounds 1 and 2 at any positions lacking a consensus and then incorporated the PRR extension, affording the 225-3 miniprotein (Fig. 1, *B* and *C*).

### Miniproteins bind Ras with nanomolar affinity and block effectors

**Figure 2.**
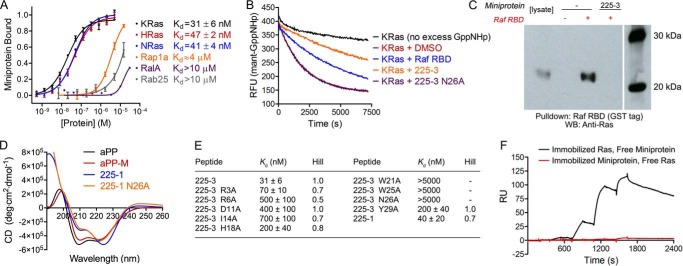
**Biochemical characterization of Ras-binding miniproteins.** *A*, fluorescence polarization of FITC-labeled 225-3 miniprotein with Ras proteins and Ras family members. All proteins are loaded with GppNHp, and all contain the G12V mutation except for RalA and Rab25 (wildtype). Dissociation constant (*K_d_*) values are mean ± S.E. for experiments performed in triplicate. *B*, dissociation of mant-labeled GppNHp from KRas(G12V) in the presence of 2 μm ligand, initiated by the addition of 1 mm GppNHp. *C*, pulldown of endogenous Ras from Capan-1 pancreatic adenocarcinoma lysate by GST-labeled B-Raf RBD after incubation with or without miniprotein. B-Raf RBD and associated proteins were isolated with glutathione beads and separated by SDS-PAGE prior to transfer and Western blotting (*WB*) with a pan-Ras antibody. The molecular weight ladder lane is spliced to conserve space. *D*, CD of purified miniproteins at 20–50 μm concentration in 50 mm sodium phosphate, pH 8. *E*, *K_d_* values of alanine mutagenesis peptides with KRas(G12V)-GppNHp determined as in *A. F*, SPR of free Ras (or miniproteins) that was flowed over a biotin CAPture sensor chip onto which biotinylated miniprotein (or Ras) (respectively) was immobilized. The experiment was performed in single-cycle format with injections at increasing concentration of free analyte. All experiments were performed at least three times. *deg*, degrees; *RU*, resonance units; *RFU*, relative fluorescence units.

To gain preliminary insight into the binding mechanism of these miniproteins, we tested the binding of yeast-displayed 225-3 to either KRas or KRas premixed with the Ras-binding domain (RBD) of Raf, a canonical Ras effector (8). The presence of the Raf RBD led to a decrease in KRas-miniprotein binding (Fig. 1*B*), suggesting that 225-3 might compete with effectors for KRas. Encouraged by this finding, we sought to biochemically characterize the KRas-miniprotein binding interaction in detail using recombinantly produced miniproteins and Ras proteins containing the oncogenic G12V mutation and bound to GppNHp, a nonhydrolyzable analog of GTP, unless otherwise noted. 225-3 bound to KRas, HRas, and NRas in solution with midnanomolar affinity (Fig. 2*A*). To determine whether 225-3 bound specifically to the Ras proteins, we tested binding to Rap1a, a GTPase whose effector domain is similar enough to Ras that it can bind the Raf RBD (8), and RalA and Rab25, two other GTPases in the Ras superfamily. The 225-3 miniprotein bound these proteins with an affinity reduced at least ∼100-fold relative to that for KRas (Fig. 2*A*).

To further characterize the KRas-miniprotein interaction, we performed nucleotide dissociation assays using the mant-GppNHp reporter (25) and found 225-3 to slow the rate of dissociation as is the case for Ras effectors such as the Raf RBD (Fig. 2*B*). This effect was not observed for the N26A mutant, which does not bind KRas (Fig. 2, *D* and *E*). To confirm that the 225-3 miniprotein could bind endogenous Ras expressed in human cancer cells, we performed pulldown assays in Capan-1 pancreatic adenocarcinoma cell lysates, capturing Raf RBD and assessing Ras-Raf binding by Western blotting. The Raf RBD pulled down Ras, and this interaction was inhibited in the presence of 225-3 (Fig. 2*C*). Curiously, we found that Ras-miniprotein binding could be detected by surface plasmon resonance (SPR) when biotinylated Ras was immobilized onto the streptavidin sensor chip and free miniprotein was flowed over it but not when biotinylated miniprotein was immobilized and free Ras was flowed over it (Fig. 2*F*).

**Figure 3.**
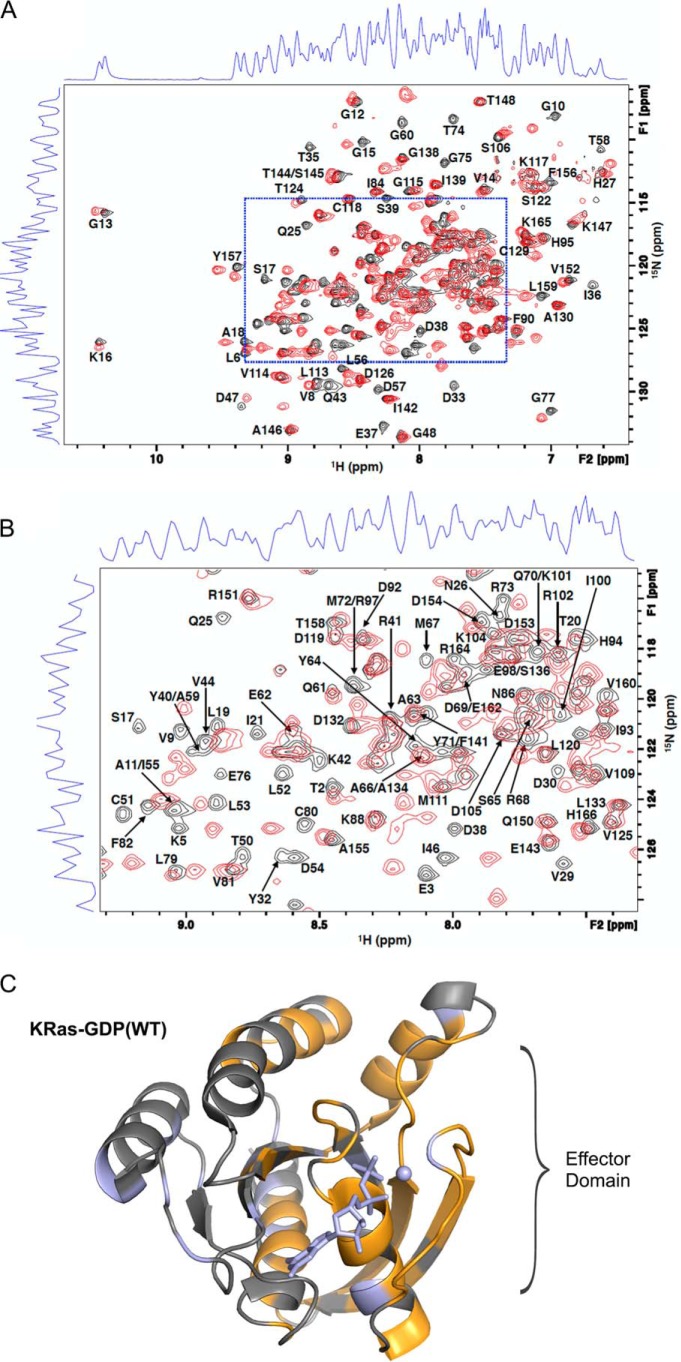
**Miniproteins bind the Ras effector domain in solution.** *A*, ^1^H-^15^N TROSY correlation data recorded for a sample of ^15^N-labeled KRas-GDP(WT) in the absence (*black*) or presence (*red*) of the 225-1 miniprotein. KRas cross-peaks were identified by comparison with published assignments (26) and used to determine significant chemical shift perturbations on a per-residue basis. *B*, zoom of the *dashed blue boxed* region from *A*. A majority (147 of 161) of the observable residues were identified (excluding the non-observable N terminus and four prolines). Overlap for 15 residues precluded either assignment and/or determination of chemical shift perturbation status. *C*, mapping of the chemical shift perturbation data on the structure of KRas-GDP(WT) (Protein Data Bank code 4OBE). Residues with significant chemical shift perturbation effects are colored *orange*, non-perturbed residues are *gray*, and proline/undetermined residues are *light blue*. An extensive chemical shift perturbation pattern was observed centered on the switch regions and supporting secondary structure elements. Minor chemical shift perturbation effects were observed in distal regions of the structure; collectively, the data are consistent with a remodeling of the Ras effector–binding region.

To map the specific miniprotein-binding site on KRas, we performed ^1^H-^15^N heteronuclear single quantum correlation (HSQC) NMR spectroscopy on ^15^N-labeled KRas(WT)-GDP, chosen because of the availability of published assignments (26), in the presence or absence of unlabeled 225-1 miniprotein, which was used in place of 225-3 for solubility reasons (Fig. 3, *A* and *B*). Comparison of shifted cross-peaks with the published assignments revealed that the residues perturbed by miniprotein binding were clustered at the Ras effector domain (Fig. 3*C*). In sum, these data demonstrated that the 225 miniproteins bound to the Ras effector domain and competed directly with effectors and that these binding properties were preserved in cancer cell lysates with endogenous Ras.

### Miniproteins bind Ras as a dimer

**Figure 4.**
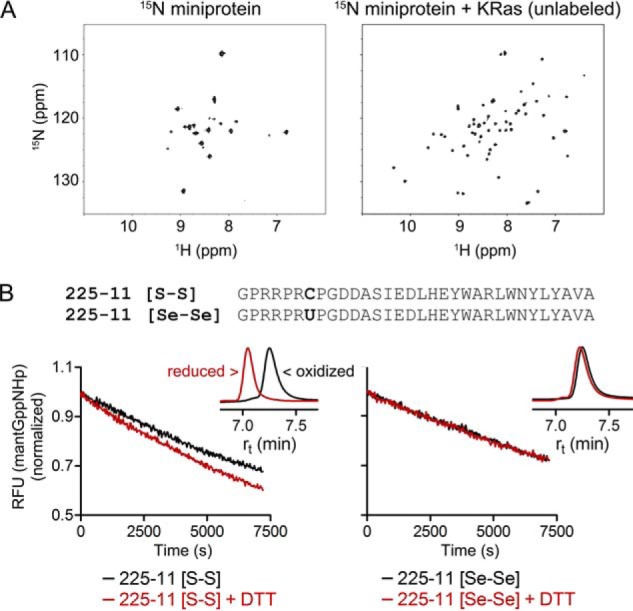
**Miniproteins bind to KRas as a homodimer.** *A*, ^1^H-^15^N heteronuclear single quantum correlation nuclear magnetic resonance spectroscopy on ^15^N-labeled 225-1 miniprotein in the absence or presence of unlabeled KRas(G12V)-GppNHp protein. The 225-1 miniprotein is used in place of 225-3 due to limiting solubility. *B*, sequence of 225-11 miniproteins and dissociation of mant-GppNHp from KRas(G12V) in the presence of disulfide- or diselenide-bonded 225-11 miniprotein dimers and in the presence or absence of 2 mm DTT as reducing agent. The *inset* shows reverse-phase HPLC of miniproteins treated with or without 10 mm DTT; the identity of reduced and oxidized species was confirmed by mass spectrometry. *U*, selenocysteine. HSQC samples were prepared and recorded once; mant dissociation was performed three times. *RFU*, relative fluorescence units.

To identify the key miniprotein residues that contribute to KRas binding, we performed an alanine mutagenesis scan of residues predicted to be on the miniprotein surface (Fig. 2*E*). Unexpectedly, every position we mutated led to a partial or full loss in KRas binding, even for residues on the PPII helix and in the loop. This suggested a more complex binding mode than a simple interaction focused around the consensus hit residues on the α-helix. We subsequently performed ^1^H-^15^N HSQC on ^15^N-labeled 225-1 miniprotein in the presence or absence of unlabeled KRas and found that the number of cross-peaks in the ^1^H-^15^N spectrum approximately doubled upon adding excess KRas to the miniprotein (Fig. 4*A*). This surprising observation could be explained either by (i) the miniprotein having two distinct binding modes, each represented approximately equally in population and in slow exchange on the NMR time scale, or (ii) a single complex being formed but containing two distinct miniprotein-Ras interfaces, *i.e.* the miniprotein binding as a dimer. In an isolated C_2_-symmetric miniprotein dimer, each residue would experience an identical environment in both protomers and would thus have a single chemical shift. Upon binding to an asymmetric target such as KRas, however, this symmetry would be broken, and at least some residues would be differentially perturbed, leading to splitting in the ^1^H-^15^N HSQC spectrum. aPP is known to form C_2_-symmetric antiparallel dimers (23, 24) but only at micromolar concentrations, and the aPP mutant used as the library scaffold contained three arginine residues in the PPII helix that were expected to disfavor dimerization via electrostatic repulsion. In contrast, a dimeric binding mode was appealing in that it provided a straightforward explanation for the puzzling intolerance to mutational substitution observed in the alanine-scanning mutagenesis (Fig. 2*E*): nearly all the residues contribute either to binding KRas or to stabilization of the miniprotein dimer. It is also consistent with the lack of Ras-miniprotein binding observed by SPR when biotinylated miniprotein was immobilized onto a sensor chip (Fig. 2*F*): as streptavidin binds biotin with femtomolar affinity, pre-immobilizing a biotinylated miniprotein dimer on a surface with excess streptavidin could wedge apart the two miniprotein protomers in a manner that would prohibit dimerization and thus abrogate KRas binding.

The dimer hypothesis also suggested an intriguing explanation for the Y7C mutant that was observed in Round 2 of the directed evolution. Tyr-7 exists at the C_2_ symmetry point in the aPP dimer (23), and because the YSD system expresses library members via the yeast secretory pathway (21), it occurred to us that cysteines at this position could be oxidized to form a disulfide bond that covalently dimerized two miniproteins. To test this, we recombinantly expressed the 225-1 Y7C miniprotein (which we named 225-11) in *Escherichia coli* and found that it spontaneously formed a disulfide-bonded dimer that bound Ras more strongly relative to the same miniprotein reduced with 2 mm dithiothreitol (DTT) (Fig. 4*B*). The 225-11 miniprotein with selenocysteine incorporated in place of cysteine (27) formed a diselenide bond that could not be reduced and that bound Ras regardless of the presence of 2 mm DTT, consistent with its significantly greater redox potential (28). Taken together, these data support the hypothesis that the 225 miniproteins bind KRas as a dimer that can be covalently stabilized by a disulfide bond between the two cysteines at position 7.

### Miniproteins capture the Ras effector domain in an open conformation

**Figure 5.**
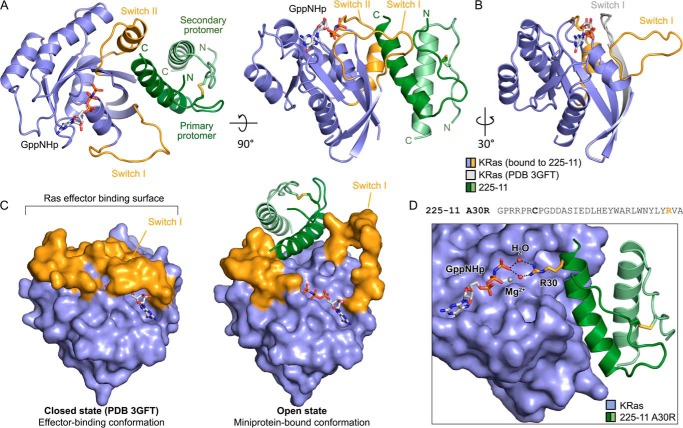
**Miniprotein homodimers bind to the KRas effector domain in an open conformation.** *A*, crystal structure of KRas(G12V)-GppNHp (*blue*) in complex with the 225-11 miniprotein (*green*) at 2.3-Å resolution. Switch I and switch II of the effector domain are shown in *orange*, inter-miniprotein disulfide bond is shown in *yellow sticks*, nucleotide is represented as *sticks*, and N and C termini are indicated for miniprotein protomers. *B*, overlay of KRas(Q61H)-GppNHp alone (*white*; Protein Data Bank code 3GFT) with the structure from *A* with miniprotein removed for clarity. *C*, structures from *A* and *B* with surface rendering of KRas. *D*, crystal structure of KRas(G12V)-GppNHp (*blue*) in complex with the 225-11 A30R miniprotein mutant (*green*) at 1.7-Å resolution. GppNHp, disulfide bond, and Arg-30 are represented as *sticks*, and coordinated waters and Mg^2+^ ion are represented as *spheres*.

**Figure 6.**
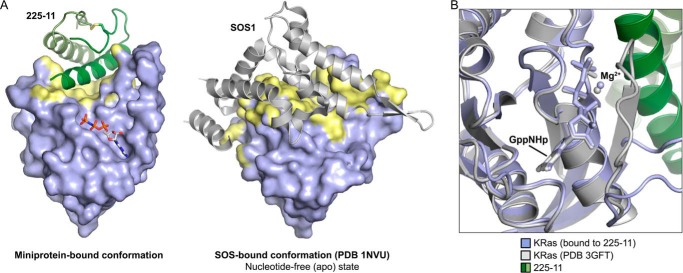
**Comparison of Ras surface and nucleotide pocket conformations.** *A*, crystal structure of KRas(G12V)-GppNHp bound to 225-11 or KRas(WT)-apo bound to SOS1. Contact residues on Ras within 4 Å of 225-11 or SOS are highlighted in *yellow. B*, close-up of nucleotide-binding pocket in KRas(G12V)-GppNHp bound to 225-11 (*blue*) aligned with KRas(Q61H)-GppNHp (*white*; PDB 3GFT). Coordinated Mg^2+^ ions are represented as *spheres*.

The non-disulfide–linked 225-1 and 225-3 miniproteins failed to afford diffraction-quality crystals with KRas, but the disulfide-linked 225-11 miniprotein afforded a crystal that enabled us to determine the structure of the KRas/225-11 complex to 2.1 Å (Fig. 5*A*). This structure revealed a disulfide-dimerized 225-11 miniprotein bound to KRas in an extended, open conformational state in which the switch I loop and β-strand 2 of the effector domain are peeled away from KRas by the invading miniprotein dimer, exposing one face of the bound GppNHp nucleotide (Fig. 5, *B* and *C*). This structure confirms direct miniprotein binding to the Ras effector domain, which is remodeled to expose an extended binding pocket (see “Discussion”). The dramatic disengagement of the switch I loop appears to be a more open version of the previously noted “state 1” conformation of Ras (29–31) and that resembles the effect of the Ras guanine nucleotide exchange factor Son of Sevenless (SOS) upon binding Ras (32) (Fig. 6*A*). The KRas-miniprotein contacts occur primarily through the α-helix of one miniprotein protomer (“primary protomer”) and β-strands 1–3 and switch II of KRas. Key miniprotein-Ras contacts appear to include miniprotein residues Glu-15, His-18, Glu-19, Trp-21, Trp-25, Asn-26, and Tyr-29 of the primary miniprotein protomer along with His-18 and Trp-21 from the “secondary” miniprotein protomer. The miniprotein dimer itself appears to be stabilized by the disulfide bond and by hydrophobic packing at the interface between protomers. The structure is broadly consistent with the alanine scan binding data (Fig. 2*E*), which showed a substantial decrease in Ras affinity for miniproteins lacking central Ras-binding residues such as Trp-21, Trp-25, and Asn-26 and a moderate decrease in affinity for more peripheral Ras-binding residues and residues involved in miniprotein-miniprotein interactions.

In light of the miniprotein-bound conformation adopted by Ras and the similarity in nucleotide conformations in miniprotein-bound and unbound Ras (Fig. 6*B*), our prior finding that nucleotide dissociation is decreased upon 225-3 miniprotein binding is surprising: the displacement of the switch I loop and the resultant exposure of the nucleotide to solvent would be expected to significantly *increase* dissociation. The mechanistic basis of this puzzling phenomenon is a subject of ongoing research in our laboratory.

### Miniprotein mutants with improved nucleotide state selectivity

**Figure 7.**
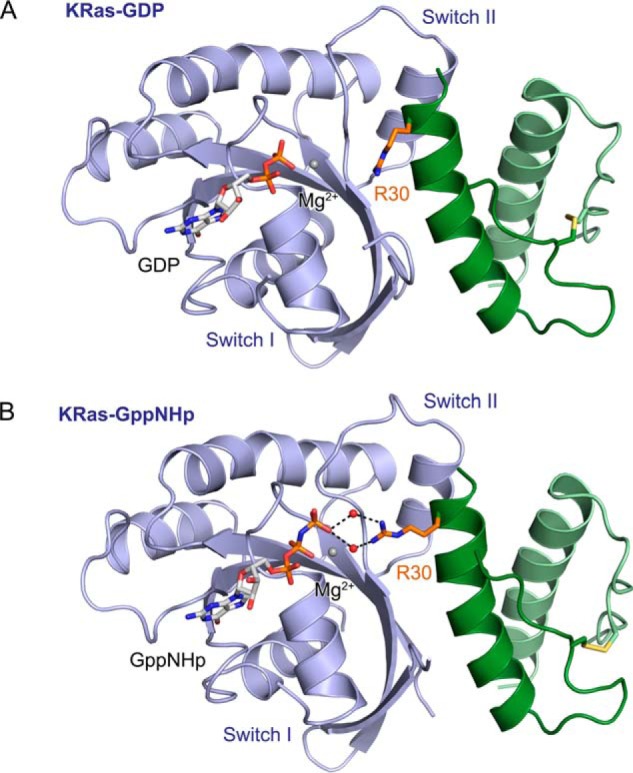
**Comparison of 225-11 A30R miniprotein bound to KRas-GppNHp *versus* KRas-GDP.** *A*, crystal structure of 225-11 A30R miniprotein (*green*) bound to Ras-GDP (*blue*) at 2.2-Å resolution. *B*, crystal structure of 225-11 A30R miniprotein (*green*) bound to Ras-GppNHp (*blue*) at 1.7-Å resolution. Switch I was partially disordered in both structures. GDP/GppNHp, disulfide bond, and *R*30 are represented as *sticks*, coordinated waters and Mg^2+^ ion are represented as *spheres*, and *dashed lines* indicate distances less than 3 Å.

The 225-11 miniprotein did not possess significant nucleotide selectivity, which was consistent in a general sense with the crystal structure of 225-11 bound to KRas-GppNHp as the nucleotide-responsive switch I loop was dislodged and the miniprotein did not come into close proximity to the nucleotide. As oncogenic Ras signaling is mediated by the GTP-bound state, we asked whether we could improve the nucleotide selectivity of the 225-11 miniprotein to favor binding to KRas-GppNHp. We prepared a scanning mutagenesis library that systematically randomized pairs of adjacent amino acids throughout the miniprotein sequence and then performed YSD with orthogonally labeled KRas-GppNHp and KRas-GDP targets. After two rounds of sorting for library members with improved selectivity for KRas-GppNHp, the screen afforded a population with a consensus mutation of A30R, located at the miniprotein residue closest to the γ-phosphate in the KRas/225-11 structure. 225-11 A30R bound more strongly to both KRas-GppNHp and KRas-GDP but with a 2-fold overall improvement in selectivity for KRas-GppNHp (see Fig. 11*B*). The crystal structure of 225-11 A30R bound to KRas-GppNHp revealed that the mutant arginine residue of the primary miniprotein protomer forms hydrogen bonds with two bound water molecules that solvate the γ-phosphate (Figs. 5*D* and 7*B*). GDP lacks the γ-phosphate, and Arg-30 is disengaged from the nucleotide in the KRas-GDP/225-11 A30R structure (Fig. 7*A*); thus, this interaction likely contributes to the improved nucleotide selectivity of this miniprotein mutant.

### Miniprotein heterodimers with picomolar affinity for Ras

**Figure 8.**
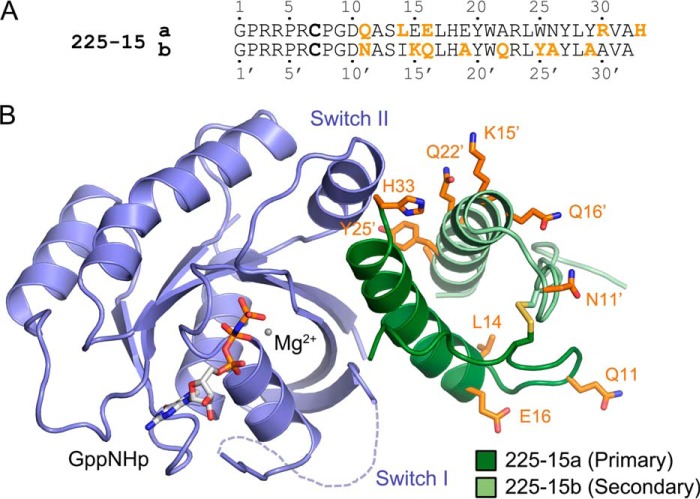
**Sequence and structure of a miniprotein heterodimer that binds Ras with improved affinity.** *A*, sequence of heterodimeric miniprotein isolated from YSD screen. Mutated residues relative to 225-11 are shown in *orange. B*, crystal structure of KRas(G12V)-GppNHp (*blue*) in complex with the 225-15a/b miniprotein (225-15a protomer in *dark green*; 225-15b protomer in *light green*) at 1.7-Å resolution. Mutated residues compared with 225-11 are shown in *orange sticks*. Switch I is partially disordered in this structure.

We then asked whether the 225-11 A30R miniprotein could be further engineered to bind KRas with higher affinity. In light of the homodimeric nature of the miniproteins, we reasoned that the miniprotein residues must have been constrained by symmetry during the directed evolution such that the most favored amino acid at each position was the best compromise between the two sites within the dimer. As KRas is an asymmetric target that forms markedly different interactions with the two miniprotein protomers, we hypothesized that breaking the symmetry and permitting each protomer to evolve independently might afford improvements in binding. To test this, we used a dual-display YSD system in which two miniproteins based on 225-11 A30R (primary protomer a and secondary protomer b) were codisplayed on the yeast surface, inspired by prior work by Boder and co-worker (33). After randomizing selected residues in both miniproteins, screening for binding at low-nanomolar concentrations of KRas-GppNHp, and merging consensus mutations, we identified a heterodimeric miniprotein, 225-15a/b, with 13 mutations overall across both protomers relative to the 225-11 A30R parent (Fig. 8*A*). The 225-15a/a homodimer bound KRas-GppNHp with ∼3-fold reduced affinity compared with 225-11 A30R, and the 225-15b/b homodimer had no detectable binding. However, the 225-15a/b *hetero*dimer bound KRas-GppNHp ∼10-fold more strongly than 225-11 A30R with an affinity of 60 ± 20 pm, which to our knowledge is more than an order of magnitude stronger than all known Ras effector proteins or synthetic ligands (see Fig. 11, *A* and *B*). 225-15a/b binds with subnanomolar affinity to all three Ras isoforms (KRas, NRas, and HRas) and to both wildtype KRas as well as the oncogenic mutants G12V, G13D, and Q61H. 225-15a/b also preserves selectivity for Ras over other GTPases with relatively weak (>100 nm) affinity for Rap1a, RalA, and Rab25 (see Fig. 11*B*). We crystallized 225-15a/b with KRas-GppNHp, which revealed a slight shift in miniprotein orientation relative to KRas and several putative contacts formed between Ras and mutated residues (Fig. 8*B*). Nearly all of these mutations occur outside of the “core” region of Ras-miniprotein contacts observed for 225-11. Further studies will be required to elucidate the individual contributions of these mutations to improved KRas binding.

### A Ras mutant stabilized in the open state

**Figure 9.**
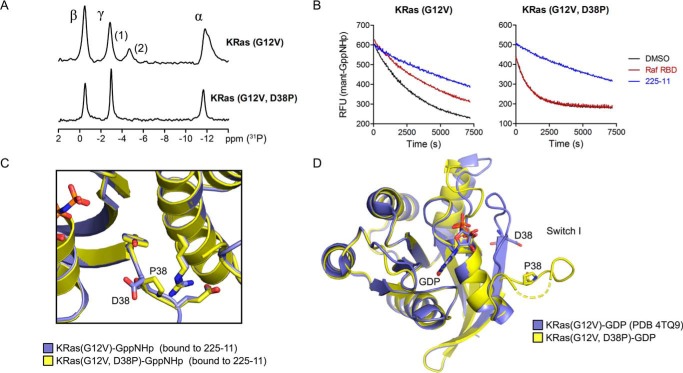
**The D38P mutant stabilizes KRas in an open conformation.** *A*, ^31^P NMR spectra of GppNHp-bound Ras mutants to determine switch I dynamics. Peaks are assigned based on prior studies of HRas (34). *B*, dissociation of mant-GppNHp from KRas(G12V) or KRas(G12V,D38P) in the presence of 2 μm ligand, initiated by the addition of 1 mm GTP. *C*, aligned structures of KRas(G12V)-GppNHp at 2.1-Å resolution and KRas(G12V,D38P)-GppNHp at 1.9-Å resolution (both bound to 225-11) with residue 38 and nearby side chains shown as *sticks. D*, aligned structures of KRas(G12V)-GDP (Protein Data Bank code 4TQ9) and KRas(G12V,D38P)-GDP at 1.6-Å resolution with Asp-38 and Pro-38 shown as *sticks*. Switch I is partially disordered for KRas(G12V,D38P)-GDP. NMR spectra were recorded once (∼48-h collection each); mant dissociation assays were performed three times. *RFU*, relative fluorescence units.

Lastly, we sought to identify a Ras mutant that would stabilize the protein in the “open” conformation trapped by our miniproteins. Such a mutant would facilitate biochemical studies of this conformational state and could be used as a target for small-molecule or fragment-based screening programs to identify molecules that engage it. Upon inspection of the Ras-miniprotein structures, we hypothesized that the open state might be stabilized by destabilizing the intact β-sheet of the “closed” state via insertion of a proline into β-strand 2, thereby mimicking the strand displacement that occurs upon miniprotein binding. We subsequently found that insertion of a proline in place of Asp-38 (Fig. 9*C*) afforded a Ras mutant that could be solubly expressed but appeared to exist exclusively in the open state.

The effector domain of KRas(G12V) exists in two conformational states that each possess distinct ^31^P NMR shifts for the γ-phosphate of the bound nucleotide, referred to as open or state 1, which cannot bind effector proteins, and closed or “state 2,” which is capable of binding effector proteins and possesses a larger upfield ^31^P shift (29, 34). Whereas KRas(G12V) exhibits a roughly 2:1 ratio of state 1:state 2, KRas(G12V,D38P) has no apparent state 2 population (Fig. 9*A*), and whereas KRas(G12V) can bind both the 225 peptides and Raf RBD, KRas(G12V,D38P) can only bind the 225 peptides (Fig. 9*B*). We note that as Asp-38 interacts with Raf (35), it is also possible that the lack of observed Raf RBD association is a consequence of disruption of the Ras-Raf binding. As the 225 miniproteins engage the open conformation of Ras, such a mutant would be expected to increase the rate of Ras-miniprotein binding by reducing the conformational rearrangement necessary for association. Indeed, the *k*_on_ between KRas(G12V,D38P) and 225-11 is 2.3-fold higher than for KRas(G12V), consistent with the D38P mutant favoring a more open conformation than the previously studied T35A mutant (29), which has also been reported to stabilize Ras in state 1 but does not exhibit an increased on-rate for miniprotein binding (see Fig. 11*B*). The increased association rate of the D38P mutant seems unlikely to result from differences in the bound state as the crystal structure of KRas(G12V,D38P) bound to 225-11 shows little difference in contacts or conformation compared with KRas(G12V) (Fig. 8*C*). We were unable to crystallize KRas(G12V,D38P) bound to GppNHp because of hydrolysis of the nucleotide analog under crystallization conditions; nevertheless, we determined the structure of KRas(G12V,D38P) bound to GDP, and this structure revealed Ras in an open conformation with a disengaged switch I loop (Fig. 8*D*). In summary, these data indicate that the D38P mutant stabilizes Ras in the open conformation that is observed in our Ras-miniprotein crystal structures.

## Discussion

The perceived “undruggability” of Ras has led to tremendous interest in new targeting approaches (1, 12). Many of these have focused on exploiting structural features of the protein, including computational screens against Ras structures that reveal induced surface pockets (13–15) and covalent targeting of the G12C mutation at the effector domain (17, 18). Our data reveal a previously unobserved Ras binding mode wherein a miniprotein dimer disrupts the switch I loop and β-strand 2 of Ras and engages β-strands 1–3 and switch II, primarily through a single miniprotein α-helix. Our data also indicate that nucleotide-bound Ras exists, if transiently, in a significantly more open conformation than had been previously observed in crystal structures of state 1 of Ras (29–31) that is similar to the structure of the nucleotide-free Ras apoprotein in complex with the SOS exchange factor (32).

**Figure 10.**
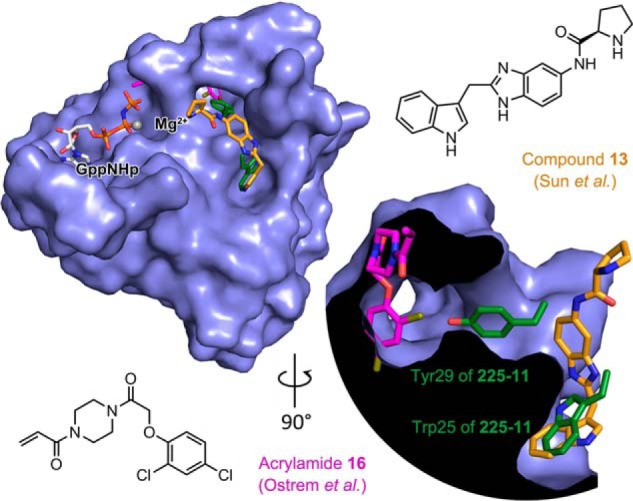
**Miniprotein binding remodels the Ras effector domain.** Surface representation of KRas(G12V)-GppNHp bound to 225-11 and 90° rotation with cutaway to illustrate the extended pocket is shown. Miniprotein is removed for clarity, and miniprotein residues Trp-25 and Tyr-29 are shown as *green sticks*. This structure was aligned with KRas bound to Acrylamide 16 (17) (Protein Data Bank code 4M22) and KRas bound to Compound 13 (15) (Protein Data Bank code 4EPY) with the corresponding Ras surface removed and Ras-binding ligands colored in *magenta* or *orange*, respectively. Portions of Acrylamide 16 overlap with KRas density from the 225-11 complex structure because Acrylamide 16 induces a larger pocket behind switch II.

These structures expose an extended groove along the Ras surface that was previously occupied by the switch 1 loop and β-strand 2. The exposed groove is partially occupied by the C-terminal α-helix of the primary miniprotein protomer with Tyr-29 binding to an induced pocket between switch II and β-strand 3 of KRas and Trp-25 binding to a shallow surface pocket among other interactions. Interestingly, the covalent KRas(G12C) inhibitors developed by Shokat and co-workers (17) bind an induced pocket on the other side of switch II from Tyr-29, and several groups have reported small-molecule ligands that bind to the same site as Trp-25 (Fig. 10*A*) (13–15). These data demonstrate that these previously isolated pockets can, in fact, be contiguous with one another. The observation of this extended pocket has significant implications for the development of small-molecule Ras inhibitors with sufficient potency for clinical use as the identification of such molecules has been hindered by the lack of sizeable binding pockets on the Ras surface.

Although the 64-amino acid miniprotein dimers are comparable in size with Ras effector proteins and cannot efficiently enter cells, they may serve as a starting point for the development of direct Ras antagonists. Most of the Ras contacts occur via the 18-residue α-helix of the primary miniprotein protomer, and in principle it should be possible to minimize this interface using an α-helical stabilization strategy such as miniprotein “stapling” (36, 37). By virtue of their unique binding mode and established competition with Ras effectors, the 225 miniproteins present an attractive basis for the development of stapled Ras inhibitors, or similar peptidomimetic structures, that directly compete with effector proteins. These miniproteins could also be implemented as genetic tools for studying the *in vivo* consequences of Ras inhibition by ectopic expression in cancer cell lines or genetically engineered animal models as has been done for shRNA knockdown of Ras expression (11). As these miniproteins bind with high affinity to all the Ras isoforms, nucleotide states, and mutants tested, further optimization will be required to develop miniproteins or derived inhibitors that are capable of specifically targeting active or oncogenic Ras. Such improved versions may be identified via counterscreening strategies such as that used to identify the KRas-GppNHp–selective A30R miniprotein mutant in this work or the recently reported KRas(G12D)-selective miniprotein based on the Sso7d scaffold (38). Although miniprotein binding appears relatively selective for Ras proteins compared with other GTPases, a similar approach may be required to reduce the midnanomolar binding of miniproteins for Rap1a.

**Figure 11.**
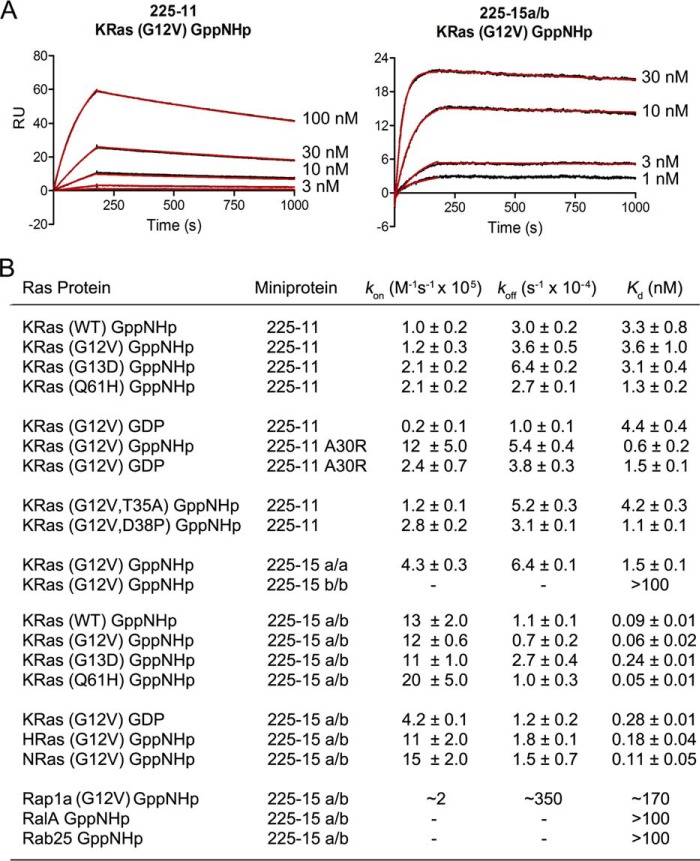
**Ras-miniprotein binding data.** *A*, representative SPR sensorgrams. For 225-15a/b, less Ras was immobilized to minimize mass transport limitations, and *k*_off_ values were only determined from the 3, 10, and 30 nm injections due to an insufficient drop in signal at 1 nm. Labels indicate injected concentration of miniprotein. *B*, SPR binding parameters determined on a Biacore X100 with a Biotin CAPture kit and biotinylated proteins. Miniproteins were injected at 3, 10, 30, and 100 nm (1, 3, 10, and 30 nm for 225-11 A30R and 225-15a/b). Values are mean ± S.E. of three independent replicates; statistics shown are calculated for *k*_on_, *k*_off_, and *K_d_* independently.

In summary, we identified a class of miniproteins from a naïve library that bind to the Ras proteins and directly antagonize Ras-effector interactions, which are essential for Ras signaling in the cell. We engineered these miniproteins to possess improved selectivity for the active, GppNHp-loaded form of Ras and evolved their binding affinity into the midpicomolar range (Fig. 11). These miniproteins should prove useful as biochemical tool compounds and in genetic models and may serve as the basis for peptidomimetic Ras inhibitors. High-resolution crystal structures demonstrate that the miniproteins bind Ras as a dimer and interact via a distinct binding mode that captures the Ras effector domain in an open conformational state, which can be stabilized by insertion of a proline at Asp-38 in Ras. Importantly, this binding mode connects two previously identified small-molecule pockets on the Ras surface, revealing a single, extended pocket. These findings present opportunities for the development of direct-acting miniprotein and small-molecule Ras inhibitors.

## Experimental procedures

### Materials and general methods

The yeast surface display vector and strain were a gift from K. D. Wittrup at the Massachusetts Institute of Technology, the Sfp expression construct was a gift from C. T. Walsh at Harvard University, and the BL21 CysE expression strain was a gift from M.-P. Strub at National Heart, Lung, and Blood Institute, National Institutes of Health. Semipreparatory high-performance liquid chromatography (HPLC) was performed with an Agilent 1200 series instrument equipped with Agilent or Supelco 250 × 10-mm C_18_ columns using a 10–100% gradient of acetonitrile in water with 0.1% trifluoroacetic acid (TFA). Liquid chromatography-mass spectrometry (LC/MS) was performed with an Agilent 1260 series instrument equipped with an Agilent 150 × 2.1-mm C_18_ column connected to an Agilent 6100 series quadrupole mass-selective detector. Gel filtration and desalting were performed on an ÄKTA FPLC (Amersham Biosciences/GE Healthcare). Flow cytometry was performed with either a Beckman Coulter MoFlo Legacy or BD FACSAria IIu.

### Purification of Ras proteins

Ras proteins (residues 1–187) were recombinantly expressed in *E. coli* BL21 Rosetta I pLysS cells from pET-derived expression vectors (Novagen) with C-terminal His_6_ and yBBr (39) tags. The cells were induced at an *A*_600_ of 0.7 with 0.3 mm isopropyl β-d-1-thiogalactopyranoside (IPTG) for 5 h at 30 °C, then harvested, and resuspended in 50 mm Tris, pH 7.5, 300 mm NaCl, 10 mm imidazole, 5 mm MgCl_2_ prior to snap freezing. For purification, the pellet was thawed, lysed with a tip sonicator, and pelleted at 30,000 × *g* for 30 min, and then the supernatant was purified with HisPur cobalt resin (Thermo Pierce), eluting with 150 mm imidazole. The protein was concentrated in a Centriprep YM-10 (Millipore) to 2 ml and purified by gel filtration on a Superdex 75 10/300 column (GE Healthcare) into 50 mm Tris, pH 7.4, 100 mm NaCl, 5 mm MgCl_2_, 1 mm DTT. For long-term storage, proteins were concentrated to >100 μm, mixed with glycerol to 10%, snap frozen, and stored at −80 °C. Rap1a, RalA, and Rab25 were expressed and purified following the same protocol. For enzymatic nucleotide exchange to GppNHp (25), the Ras protein was exchanged by gel filtration into 32 mm Tris, pH 8, 200 mm (NH_4_)_2_SO_4_, 1 mm DTT, 0.5 mm NaN_3_, 1 μm ZnCl_2_. The protein was concentrated to >100 μm and then mixed with GppNHp or GppCp to 0.5–1.0 mm followed by 10–20 units of calf intestinal alkaline phosphatase (New England Biolabs). The protein was incubated at room temperature for 30 min, then MgCl_2_ was added to 5 mm, and the protein was gel-filtered as before. Nucleotide state was verified by reverse-phase HPLC (C_18_ column) using a 20-min isocratic run in 100 mm potassium phosphate, pH 6.5, 10 mm tetrabutylammonium bromide, 7.5% acetonitrile. Elution times for GDP, GTP, and GppNHp were determined with pure nucleotide standards (Sigma-Aldrich).

### Purification of B-Raf RBD

The Raf RBD was recombinantly expressed in *E. coli* BL21 Rosetta I pLysS cells from a pGEX-3 vector (GE Healthcare) with an N-terminal glutathione *S*-transferase (GST) tag. The cells were grown and induced as above for Ras, then harvested, and resuspended in lysis buffer (PBS + 1 mm DTT + 0.5 mm EDTA) prior to snap freezing. For purification, the pellet was thawed, lysed, and clarified as above for Ras, and then the clarified lysate was purified with immobilized glutathione resin (Thermo Pierce), eluting with 50 mm Tris, pH 8.0, 10 mm reduced glutathione, 1 mm DTT, 0.5 mm EDTA. The sample was concentrated, purified by gel filtration, and stored as described for Ras.

### Protein labeling by Sfp phosphopantetheinyl transferase

Purified Ras was gel-filtered on a Superdex 75 10/300 column into 64 mm Tris, pH 7.5, 5 mm MgCl_2_, 1 mm DTT. Ras (50–150 μm) was mixed with 1.0–1.3 eq of coenzyme A ligated to biotin-maleimide or Alexa Fluor 647-maleimide followed by Sfp to 3–5 μm. The reaction was incubated at room temperature for 1 h, then gel-filtered as described above, and verified by MALDI/MS or absorbance spectroscopy. Sfp was purified as reported (39). The yBBr tag was located C-terminal to the hypervariable region and hexahistidine tag on the C terminus of full-length Ras proteins. Assays performed with unlabeled and/or untagged Ras proteins and endogenous, untagged Ras in human cancer cell lysates (*e.g.* Figs. 2–4) established that miniprotein-Ras binding was not an artifact of yBBr tagging.

### Yeast surface display

Yeast display protocols were generally carried out as described by Wittrup and co-workers (21, 40). DNA libraries were prepared by overlap extension of synthetic primers (Eurofins MWG Operon, Integrated DNA Technologies, or Yale Keck Center). Randomized positions were either NNK codons or trimer phosphoramidites lacking cysteine (Glen Research). For generation of mutagenized library templates using error-prone PCR, the method of Zaccolo *et al.* (41) utilizing dPTP and 8-oxo-dGTP was used as described in Chao *et al.* (40). Libraries were sorted by magnetic-activated cell sorting (MACS) and/or by FACS. In general, yeast were induced overnight with galactose-containing growth medium, then suspended at ∼10^8^ cells/ml in PBS containing 0.1 mg/ml bovine serum albumin (BSA), and incubated with recombinant KRas(G12V) labeled with either biotin or Alexa Fluor 647 using Sfp (39). Labeled cells were isolated either with an AutoMACS instrument (Miltenyi) or by FACS (MoFlo Legacy). Library members were sequenced by purifying yeast DNA with a miniprep kit (Qiagen), transforming into *E. coli*, and Sanger sequencing isolated colonies. For dual-target FACS, Alexa Fluor 647-labeled Ras-GppNHp and biotin-labeled Ras-GDP were mixed with cells, and Ras-GDP was imaged with streptavidin-conjugated R-phycoerythrin. For dual-miniprotein display, two separate miniprotein ORFs were expressed off each direction of the Gal1/10 promoter as described by Jiang and Boder (33).

### Production of 225-1 and 225-3 miniproteins

**Figure 12.**
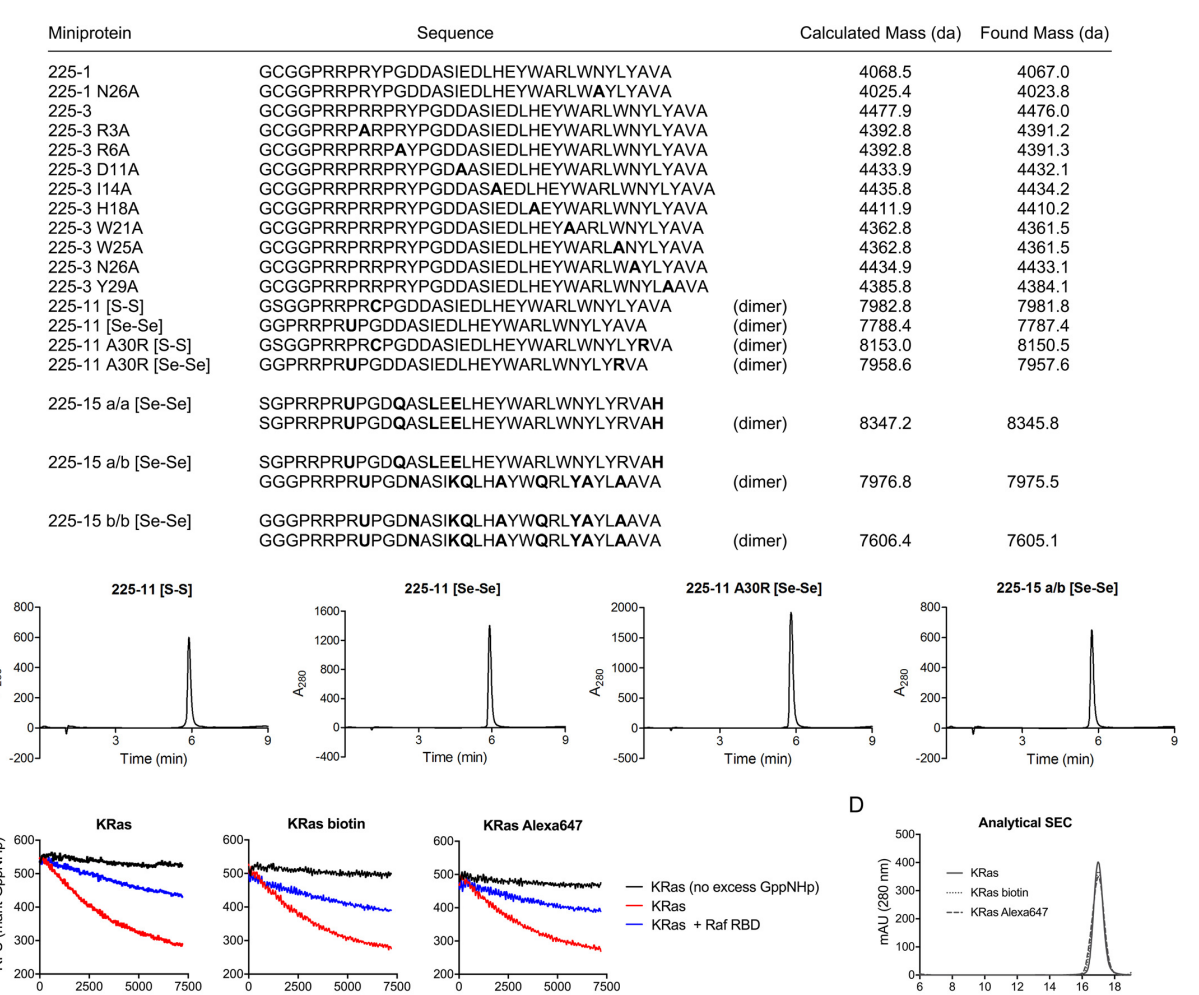
**Sequences and masses of miniproteins used in this work and analysis of labeled Ras proteins.** *A*, sequences and calculated/found (electrospray MS, positive mode) masses of recombinantly expressed miniproteins. *U*, selenocysteine. All dimers are oxidized. *B*, HPLC traces (*A*_280_) of representative miniproteins following purification. *C*, dissociation of mant-labeled GppNHp from KRas(G12V) proteins in the presence or absence of 5 μm Raf RBD, initiated by the addition of 1 mm GppNHp. *D*, size-exclusion chromatography of KRas(G12V) proteins (Superdex 200 Increase 10/300 GL column; void volume is at ∼7 ml). *RFU*, relative fluorescence units.

Miniproteins can be prepared either by recombinant expression in *E. coli* or by chemical synthesis. All miniproteins described in this work were prepared by recombinant expression. For chemical synthesis, see “Production of 225-11 and 225-15 miniproteins” below. For expression, the 225-1 and 225-3 miniprotein coding sequences were cloned into a pET30a vector (Novagen) and transformed into *E. coli* BL21 cells containing the pG-KJE8 chaperone plasmid (Clontech). Cells were grown in LB medium at 37 °C to an *A*_600_ of ∼0.7 and then induced with 500 μm IPTG and 200 mg of arabinose for 5 h at 30 °C. The cells were pelleted; resuspended in 50 mm sodium phosphate, pH 7.5, 1000 mm NaCl, 10 mm imidazole; and snap frozen. The pellets were lysed, clarified, and purified by cobalt affinity chromatography as for the Ras proteins except with the above resuspension buffer and 350 mm imidazole for elution. Eluted protein was concentrated to 2 ml; exchanged into 50 mm Tris, pH 8, 250 mm NaCl, 1 mm DTT, 0.5 mm EDTA using two tandem 5-ml HiTrap desalting columns (GE Healthcare); and then cleaved with tobacco etch virus protease overnight at room temperature. The cleavage reaction was desalted with Sep-Pak Classic C_18_ cartridges (Waters) and then purified by HPLC as indicated under “Materials and general methods” (Fig. 12). For labeling with fluorescein isothiocyanate (FITC), HPLC-purified miniproteins were diluted to 250–500 μm in dimethyl sulfoxide (DMSO), reduced with 1–2 eq of tris(2-carboxyethyl) phosphine, and labeled with 2 eq of *N*-(5-fluoresceinyl)maleimide for 30 min prior to purification by HPLC. Biotinylation was similarly performed with biotin-PEG_2_-maleimide (Thermo Pierce). Miniprotein solutions were quantified by UV absorbance (280 nm) in PBS using predicted extinction coefficients based on primary sequence (http://web.expasy.org/protparam/).^7^

### Production of 225-11 and 225-15 miniproteins

The 225-11 and 225-15 miniprotein coding sequences were cloned into a pET30a vector (Novagen), transformed into BL21 CysE cells, and expressed either with cysteine or selenocysteine as described (27) using double labeling minimal medium with unlabeled methionine and 10 g/liter glucose instead of glycerol and inducing at an OD of 1.5 with 200 μm IPTG for 15 min prior to pelleting, resuspending with fresh medium containing cystine or selenocystine, and growing at 25 °C overnight. The miniproteins were purified as described above for 225-1 and 225-3 except with no DTT added at any step. Heterodimeric miniproteins were expressed from bicistronic vectors; for example, the 225-15a/b miniprotein construct was prepared by digestion/ligation of the following sequence with NcoI and XhoI into pET30a: CCATGGAAAACCTGTATTTTCAGAGCGGCCCGCGTCGTCCGCGTTGCCCGGGCGATCAGGCGAGCCTGGAAGAACTGCATGAATATTGGGCGCGCCTGTGGAACTATCTGTATCGCGTGGCGCATTAAGCTAGCTGGCTGCGACTAGGAATTCAATAATTTTGTTAACTTTAAGAAGGAGATATACATATGGTCGACCACCATCATCATCATCATTCTTCTGGTCTGGTGCCACGCGGTTCTGGTGTGAAAGAAACCGCTGCTGCTAAATTCGAACGCCAGCACATGGACAGCCCAGATCTGGGTACCGACGACGACGACAAGGAGCTCGAAAACCTGTATTTTCAGGGTGGCGGCCCGCGTCGTCCGCGTTGCCCGGGCGATAACGCGAGCATTAAACAGCTGCATGCGTACTGGCAGCGCCTGTATGCGTATCTGGCGGCGGTGGCGTAACTCGAG.

Alternatively, miniproteins can be prepared using Fmoc (*N*-(9-fluorenyl)methoxycarbonyl)–based solid-phase peptide synthesis on low-loading Rink Amide MBHA resin. Double couplings are recommended following arginine and isoleucine residues and throughout the N-terminal arginine- and proline-rich region, and disulfide bonds can be formed selectively using 2,2′-dipyridyldisulfide (42).

### Circular dichroism (CD) spectroscopy

CD measurements were carried out on a Jasco J-710 spectrophotometer equipped with a PTC-348W temperature controller. Miniproteins were placed in a 1-mm quartz cuvette at 20–80 μm in 50 mm sodium phosphate, pH 8, unless otherwise noted. For spectra, CD was scanned from 260 to 190 nm at 25 °C. For melting curves, the temperature was increased from 10 to 90 °C while recording the CD at 222 nm.

### Fluorescence polarization

FITC-labeled miniproteins were diluted to 30 nm in 50 mm Tris, pH 7.4, 100 mm NaCl, 5 mm MgCl_2_, 1 mm DTT and then added to 40 μl of 2× protein stock (in the same buffer) dispensed into a 384-well black microplate (Corning). The plate was allowed to rest for 45 min at room temperature, and then fluorescence anisotropy was recorded on a SpectraMax M5 (Molecular Devices) with excitation at 485 nm, emission at 525 nm, and cutoff at 515 nm. Each concentration point was prepared in triplicate, and each well was read twice and averaged. Data were plotted using Prism (GraphPad) and fit to a one-site–specific binding model with Hill coefficient.

### Nucleotide dissociation and hydrolysis experiments

Syntheses of mant-GppNHp and mant-GDP were performed as described by Hiratsuka (43), and mant-GppNHp and mant-GDP were loaded onto KRas as described above for unlabeled nucleotides. Assays were performed by adding 100 μl of buffer (50 mm Tris, pH 7.4, 100 mm NaCl, 5 mm MgCl_2_, 1 mm DTT) containing Raf or miniprotein to a black 96-well plate (Corning) followed by 50 μl of 2 μm mant-nucleotide–loaded Ras protein. The final DMSO concentration was 0.1%. After incubating for 30 min in the dark, unlabeled nucleotide was added, and the mant fluorescence was tracked over the course of 2 h with a SpectraMax M5 (Molecular Devices), recording six reads every 30 s (excitation, 370 nm; emission, 450 nm; cutoff, 435 nm).

### Surface plasmon resonance

Biotinylated KRas (bound to GppNHp or GDP) was diluted to 50 nm in running buffer (50 mm Tris, pH 7.4, 100 mm NaCl, 5 mm MgCl_2_, 0.5 mm EDTA, 0.005% Tween 20, 10 μm nucleotide) and immobilized on a Biotin CAPture chip in a Biacore X100 SPR system (GE Healthcare). Miniproteins were injected at 3, 10, 30, and 100 nm or at 1, 3, 10, and 30 nm for 225-11 A30R and 225-15a/b. Data were processed and analyzed with the instrument software using a one-step binding model.

### Pulldown assays in Capan-1 cell lysate

Capan-1 adenocarcinoma cells were obtained from the American Type Culture Collection (ATCC) and cultured at 37 °C in Iscove's modified Dulbecco's medium containing 20% fetal bovine serum. Cells were lysed by incubating for 10 min in 50 mm HEPES, pH 7.5, 100 mm NaCl, 10 mm MgCl_2_, 1 mm EDTA, 5 mm DTT, 1% (v/v) Triton X-100, 1× protease inhibitor (Thermo Scientific) and pelleted at 16,000 × *g* for 10 min, and then the supernatant was snap frozen and stored at −80 °C. The lysate was diluted 10-fold with detergent-free buffer and preincubated with or without miniprotein (10 μm) for 10 min before adding GST-tagged Raf RBD (5 μm) and 50 μl of glutathione-agarose beads (Thermo Scientific) and rotating for 60 min. The beads were washed three times with lysis buffer containing 0.1% (v/v) Triton X-100 and then eluted by soaking in SDS loading buffer for 5 min before pelleting at 16,000 × *g* and boiling for 5 min. Samples were run out on a 10% SDS-polyacrylamide gel, transferred to a 0.45-μm nitrocellulose membrane (Whatman), blocked for 1 h with 5% dry nonfat milk in Tris-buffered saline with 0.1 (v/v) Tween 20 (TBS/T), and then incubated overnight at 4 °C in 5% BSA in TBS/T with a 1:1000 dilution of anti-Ras rabbit mAb (Cell Signaling Technology, catalog number 3339) prior to visualization with anti-rabbit HRP conjugate (Cell Signaling Technology, catalog number 7074) and SuperSignal West Pico chemiluminescent imaging reagents (Thermo Scientific) with BioMax Light film (Eastman Kodak Co./Carestream Health) using a biotinylated ladder and anti-biotin-HRP (Cell Signaling Technology, catalog numbers 7727 and 7075).

### Nuclear magnetic resonance

To prepare ^15^N-labeled KRas or miniprotein, cells were grown in minimal medium containing 1 g/liter ^15^NH_4_Cl, expressed and purified as described above, and then exchanged into NMR buffer (50 mm HEPES, pH 7.4, 50 mm NaCl, 2 mm MgCl_2_, 2 mm tris(2-carboxyethyl)phosphine, 0.1 mm EDTA, 0.02% NaN_3_). Ras and miniprotein were mixed in a 1:2 ratio at <50 μm to avoid aggregation, then concentrated to 100–200 μm, mixed with 119 volumes of D_2_O (final concentration of 5%), filtered through a 0.45-μm membrane, and then added to a Shigemi BMS-3 NMR sample tube. HSQC NMR experiments were performed at 298 K with 256 ^15^N increments using TROSY (44) on a 700-MHz Bruker system (equipped with cryoprobe). Data were processed with NMRPipe (45) and visualized using NMR View (46). ^31^P NMR nucleotide studies were performed as described (29) at a concentration of 1 mm protein using a Varian 400-MHz NMR spectrometer.

### X-ray crystallography and structure determination

KRas(G12V)-GppNHp/225-11 complex crystallized in 0.1 m ammonium sulfate, 20–25% PEG3350. KRas(G12V)-GppNHp/225-11 A30R complex crystals were obtained in 0.2 m calcium chloride, 20% PEG3350. 225-11 and 225-11 A30R complex crystals were obtained by the sitting drop vapor diffusion method and transferred to a reservoir supplemented with 20% (v/v) glycerol followed by flash freezing in liquid nitrogen. Both KRas(G12V)-GDP/225-11 and KRas(G12V)-GDP/225-11 A30R crystals were grown by the hanging drop vapor diffusion method using a reservoir solution of 0.25 m calcium chloride, 24% PEG3350. KRas(G12V)-GppNHp/225-15a/b crystals were also grown in hanging drop fashion in 0.2 m ammonium sulfate, 26% PEG3350, 0.1 m HEPES, pH 7.5. Crystals for KRas(G12V,D38P)-GDP and KRas(G12V,D38P)-GppNHp/225-11 were grown in 0.2 m sodium fluoride, 23% PEG3350 and 0.25 m calcium chloride, 21% PEG3350, respectively. Mg^2+^ was present in all crystallization conditions as part of the KRas stock solution (2.5 mm final concentration in crystallization drops). These crystals were transferred to cryoprotectant solution that had 14–19% PEG400 added to the corresponding reservoir solution and flash frozen in liquid nitrogen prior to data collection. Diffraction data sets were collected at −170 °C at the 24-ID-C and 24-ID-E beamlines (Northeastern Collaborative Access Team) of the Advanced Photon Source. Data sets were processed with the HKL program suites. Initial molecular replacement solutions were obtained by PHASER in the CCP4 suite using the coordinate of previously determined KRas structure as a search model (Protein Data Bank code 3GFT). Complete complex models were built through iterative cycles of manual model building in Coot and structure refinement using REFMAC5 and PHENIX. The Ramachandran plots, calculated by MolProbity, contain no residues in disallowed regions for all structures. All the structure model figures in the paper were prepared using PyMOL (The PyMOL Molecular Graphics System, Version 1.3, Schrödinger, LLC.). The atomic coordinates and structure factors have been deposited in the Protein Data Bank (see Table 1 for Protein Data Bank codes).

**Table 1:** **Crystallographic data collection and refinement statistics** PDB, Protein Data Bank; APS, Advanced Photon Source; r.m.s.d., root mean square deviation; N/A, not applicable.

	KRas(G12V)-GppNHp/225-11 (PDB 5WPL)	KRas(G12V)-GppNHp/225-11 A30R (PDB 5WPM)	KRas(G12V)-GppNHp/225-15a/b (PDB 5WLB)	KRas(G12V)-GDP/225-11 (PDB 5WHA)	KRas(G12V)-GDP/225-11 A30R (PDB 5WHB)	KRas(G12V,D38P)-GppNHp/225-11 (PDB 5WHE)	KRas(G12V,D38P)-GDP (PDB 5WHD)
**Data collection**							
Source	APS 24-ID	APS 24-ID	APS 24-ID	APS 24-ID	APS 24-ID	APS 24-ID	APS 24-ID
Wavelength (Å)	0.979180	0.979180	0.979180	0.979180	0.979180	0.979100	0.979180
Space group	P 2_1_	P 2_1_	P 2_1_	P 2_1_	P 2_1_	P 2_1_	P 2_1_
Unit cell							
*a*, *b*, *c* (Å)	40.9, 234.5, 48.1	41.9, 37.8, 70.2	38.1, 86.8, 71.0	48.0, 40.3, 230.9	41.0, 232.0, 47.9	40.6, 232.5, 48.2	37.2, 80.9, 108
α, β, γ (°)	90, 90.1, 90	90, 103.4, 90	90, 103.6, 90	90, 91.0, 90	90, 90.1, 90	90, 90.2, 90	90, 90.6, 90
Resolution (Å)	50.0–2.15 (2.23–2.15)*^a^*	68.3–1.72 (1.81–1.72)	86.8–1.72 (1.81–1.72)	115.5–2.04 (2.09–2.04)	116.0–2.18 (2.24–2.18)	19.5–1.91 (2.01–1.91)	64.8–1.64 (1.67–1.64)
Unique reflections	24,546	21,613	47,613	55,395	44,361	65,457	74,937
Redundancy	3.6 (2.5)	3.0 (3.1)	3.6 (2.9)	3.0 (3.0)	2.5 (2.1)	3.3 (3.1)	3.3 (1.2)
Completeness (%)	98.1 (84.0)	98.9 (99.3)	99.3 (99.0)	97.0 (98.6)	95.3 (91.2)	95.2 (92.6)	95.8 (49.8)
*R*_merge_	0.099 (0.433)	0.090 (0.551)	0.068 (0.916)	0.125 (0.574)	0.097 (1.129)	0.083 (0.475)	0.067 (0.915)
*I*/σ	12.9 (2.1)	9.2 (1.9)	13.9 (1.2)	7.7 (1.9)	7.9 (0.7)	13.4 (3.5)	11.5 (0.7)

**Refinement**							
Resolution	50.0–2.15	68.3–1.72	69.1–1.72	115.5–2.04	116.0–2.18	19.54–1.91	108.0–1.64
No. atoms							
Protein	5,027	1,217	2,569	4,929	5,046	5,032	5,089
Peptides	2,046	533	1,081	2.045	2,117	2,092	N/A
Ligands/ions	141	39	89	124	126	142	112
Water	228	69	180	180	234	391	290
*R*_work_/*R*_free_ (%)	0.205/0.258	0.235/0.285	0.192/0.233	0.238/0.277	0.204/0.252	0.184/0.227	0.192/0.225
r.m.s.d.							
Bond lengths (Å)	0.014	0.007	0.010	0.002	0.002	0.005	0.005
Bond angles (°)	1.673	1.161	1.191	0.461	0.447	0.872	0.916
Average B-factor	26.9	36.0	32.0	30.0	47.0	27.0	27.0
Ramachandran plot favored/allowed/disallowed (%)	97.4/2.6/0.0	99.5/0.5/0.0	98.0/2.0/0.0	98.4/1.6/0.0	99.4/0.6/0.0	98.7/1.3/0.0	98.4/1.6/0.0

*^a^* Numbers in parentheses correspond to the last resolution shell.

## Author contributions

J. H. M., S. Y. S., S.-J. L., and G. L. V. designed research. J. H. M., S. Y. S., S.-J. L., P. K. S., Y. J., and M. A. D. performed research. J. H. M., S. Y. S., S.-J. L., M. A. D., and G .L. V. analyzed data. J. H. M., S. Y. S., S.-J. L., and G. L. V. wrote the paper. All authors reviewed the results and approved the final version of the manuscript.

## Footnotes

7 Please note that the JBC is not responsible for the long-term archiving and maintenance of this site or any other third party-hosted site.

## Footnotes

6 The abbreviations used are:

YSD: yeast surface display
aPP: avian pancreatic polypeptide
PPII: type II polyproline
RBD: Ras-binding domain
SPR: surface plasmon resonance
HSQC: heteronuclear single quantum correlation
SOS: Son of Sevenless
GppNHp: guanosine 5′-(β,γ-imido)triphosphate
mant: 3′-*O*-(*N*-methylanthraniloyl)
GppCp: guanosine 5′-(β,γ-methyleno)triphosphate
dPTP: 2′-deoxy-P-nucleoside 5′-triphosphate
8-oxo-dGTP: 8-oxo-2′-deoxyguanosine 5′-triphosphate
IPTG: isopropyl β-d-1-thiogalactopyranoside
TROSY: transverse relaxation-optimized spectroscopy.
